# Occurrence and Abundance of *Dermacentor reticulatus* in the Habitats of the Ecological Corridor of the Wieprz River, Eastern Poland

**DOI:** 10.3390/insects12020096

**Published:** 2021-01-23

**Authors:** Zbigniew Zając, Aleksandra Sędzikowska, Weronika Maślanko, Aneta Woźniak, Joanna Kulisz

**Affiliations:** 1 Chair and Department of Biology and Parasitology, Medical University of Lublin, Radziwiłłowska 11 st., 20-080 Lublin, Poland; aneta.wozniak@umlub.pl (A.W.); joanna.kulisz@umlub.pl (J.K.); 2 Department of General Biology and Parasitology, Medical University of Warsaw, Chałubińskiego 5 st., 02-004 Warsaw, Poland; aleksandra.sedzikowska@wum.edu.pl; 3 Department of Animal Ethology and Wildlife Management, University of Life Sciences in Lublin, Akademicka 13 St., 20-950 Lublin, Poland

**Keywords:** ecological corridors, ticks, tick activity, tick occurrence, *Dermacentor reticulatus*

## Abstract

**Simple Summary:**

Ecological corridors are zones of natural vegetation, which connect with other vegetation strips to create migration routes and provide shelter for animals. One of the longest ecological corridors in eastern Poland runs along the Wieprz River valley. We examined the occurrence and relative abundance of *Dermacentor reticulatus* in research plots established in the river valley and confirmed the presence of this tick species in each of the seven examined sites. The results of our research show that the habitats of the river ecological corridor can be regarded as preferred habitats of *D. reticulatus* in eastern Poland.

**Abstract:**

Ecological corridors are zones of natural vegetation, which connect with other vegetation strips to create migration routes for animals and plants. The aim of our study was to investigate the occurrence and relative abundance of *Dermacentor reticulatus* in various habitats of the ecological corridor of the Wieprz River in eastern Poland. Ticks were collected using the flagging method in seven sites within the ecological corridor of the Wieprz River, i.e., one of the longest uninterrupted vegetation strips in eastern Poland. The presence of *D. reticulatus* adults was confirmed in each of the examined sites. The autumn peak of tick activity dominated in most plots. During this period, on average up to 309.7 individuals were collected within 30-min. The results of our study show that, due to the high abundance of local *D. reticulatus* populations, the habitats located in the ecological corridor of the Wieprz River can be regarded as preferred habitats of this tick species.

## 1. Introduction

Ecological corridors are zones of natural vegetation located mainly along river valleys, mountain ranges, and forest complexes. They serve as migration routes for animals, plants, and fungi. By connecting with other vegetation belts, they create an uninterrupted network providing shelter for animals and enabling them to acquire food and raise their offspring [1]. They are extremely important, given the progressive fragmentation of the environment caused by human activity [2]. Although the vast majority of ecological corridors stretch along large areas that have not been changed by human activity or have been only slightly transformed, some smaller-scale ecological corridors can be identified within large conurbations [3].

The concept of ecological corridors is currently being implemented in many European countries in accordance with European legislation [4]. Ecological corridors with national, regional, and even continental importance are being established [4,5]. Several ecological corridor projects have been implemented in Poland. The first one, created in the 1990s (the National Ecological Network (ECONET-PL project), was a coherent part of the European Ecological Network (EECONET). Particular emphasis in this project was placed on ecological corridors stretching along watercourses. In the 2000s, it was modified by the fusion of ECONET-PL with the National System of Protected Areas, with particular emphasis on coherence with Natura 2000 areas [6].

**Figure 1:**
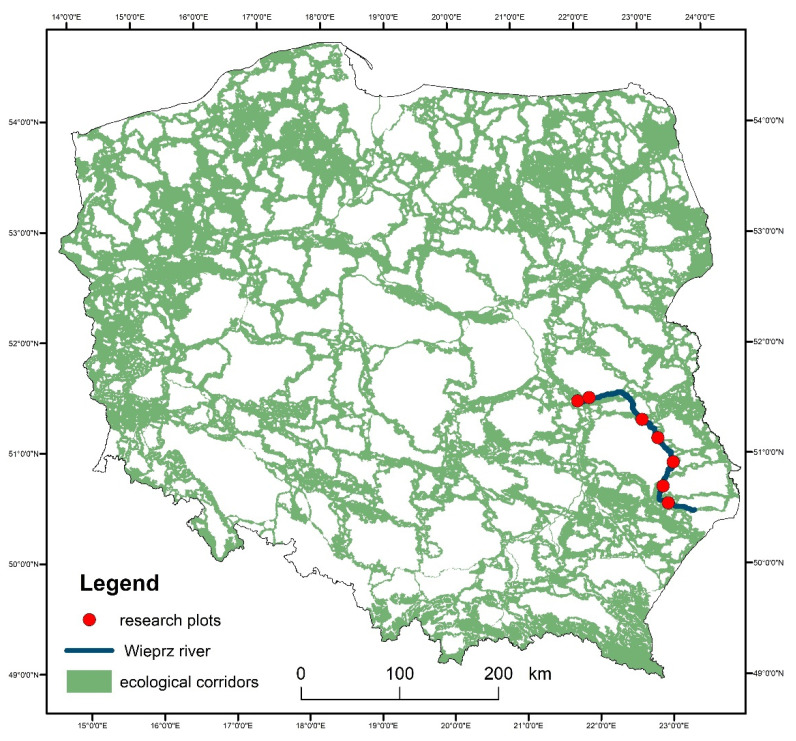
Network of ecological corridors in Poland based on the study by the Mammal Research Institute of the Polish Academy of Sciences [7]. The ecological river corridors in Poland are marked in green; the blue line marks the course of the ecological corridor in the Wieprz River valley.

In eastern Poland, three large ecological corridors have been established along river valleys, i.e., along the Vistula and Bug rivers, which are classified as routes with European importance and smaller corridor with regional importance along the Wieprz River (Figure 1).

Investigations conducted so far on the occurrence of ticks in river valleys in various parts of the world have confirmed that this type of habitat offers these arthropods favorable living conditions. *Ixodes scapularis*, *I. dammini*, *I. ricinus*, *Dermacentor andersoni*, and *D. variabilis* are some of the species that have been collected in these habitats [8,9,10,11]. Researchers have reported the presence of *D. reticulatus* in river valleys of Poland as well [12]; however, to the best of our knowledge, no simultaneous comparative studies have been carried out to date on the occurrence and abundance of this species in habitats of river valleys.

Additionally, comprehensive knowledge of the ecology and biology of *D. reticulatus* ticks is important due to their great epidemiological and veterinary importance, i.e., the role of this species as vectors of numerous pathogens [13]. The ticks transmit TBE virus, Omsk hemorrhagic fever virus, protozoa *Babesia canis*, *B. caballi*, and *Theileria equi*, bacteria *Anaplasma marginale*, and rickettsiae *Rickettsia raoultii*, *R. slovaca* [14,15,16,17]. Moreover, *R. helvetica*, *Borrelia burgdorferi* s.l., *Coxiella burneii*, *Francisella tularensis* (with an unknown vector role) have been detected in *D. reticulatus* ticks [14].

The aim of our study was to investigate the occurrence and relative abundance of *D. reticulatus* ticks in habitats located in the immediate vicinity of a river bed (the ecological corridor of the Wieprz River). The relevance of this study is supported by the increasing number of reports on the presence of *D. reticulatus* in areas where the species was not found previously, e.g., north, western, south-western, and central Poland [18,19,20]. Whereas, eastern Poland is an area of high density of *D. reticulatus* [21] however, there are no previous simultaneous studies on monitoring of seasonal activity of that species in various physico-geographical areas including influence of abiotic and biotic factors in the area of river ecological corridor.

## 2. Materials and Methods

### 2.1. Study Area

The study was carried out in the ecological corridor along the Wieprz River (Figure 1), i.e., the longest river in Lublin Province (eastern Poland) with a length of 303 km and a basin area of 10,400 km^2^ [22]. As a hydrographic axis of the region, the river valley constitutes an ecological corridor with favorable living conditions for fauna and flora and a route of undisturbed migration for animals [7,23].

**Figure 2:**
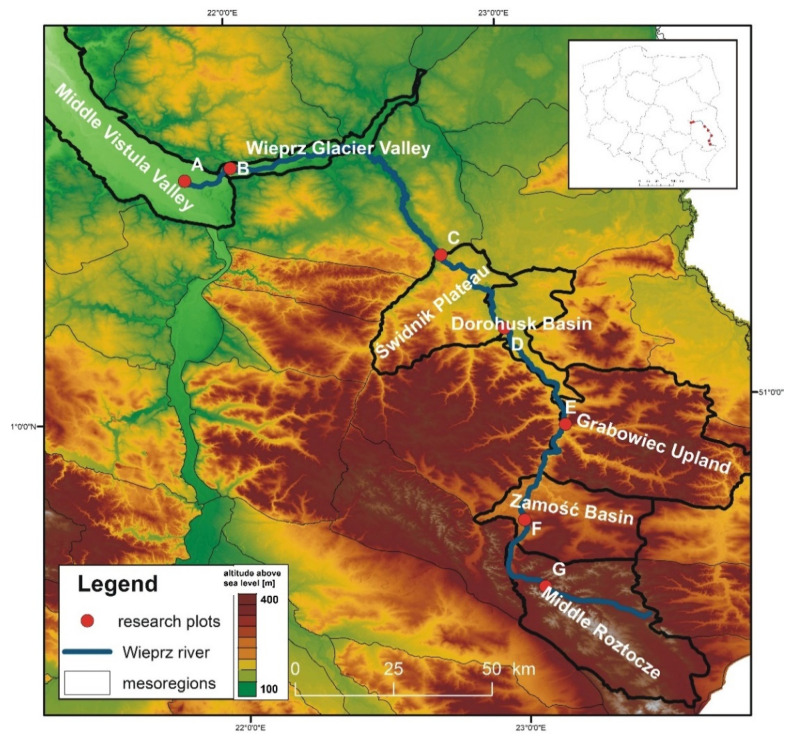
Location of the research plots (A–G). The Wieprz River is indicated by the blue line. Based on geoportal.gov.pl [26].

As shown by the physico-geographical division of Poland proposed by Kondracki [24], the Wieprz River flows through seven mesoregions differing in their geomorphological structure, water conditions, thermal conditions, and vegetation cover (Table 1). Within each of these mesoregions, one research plot (marked as A–G) (Figure 2, Table 1) was established near the river to collect ticks (see Section 2.2).

**Figure A1:**
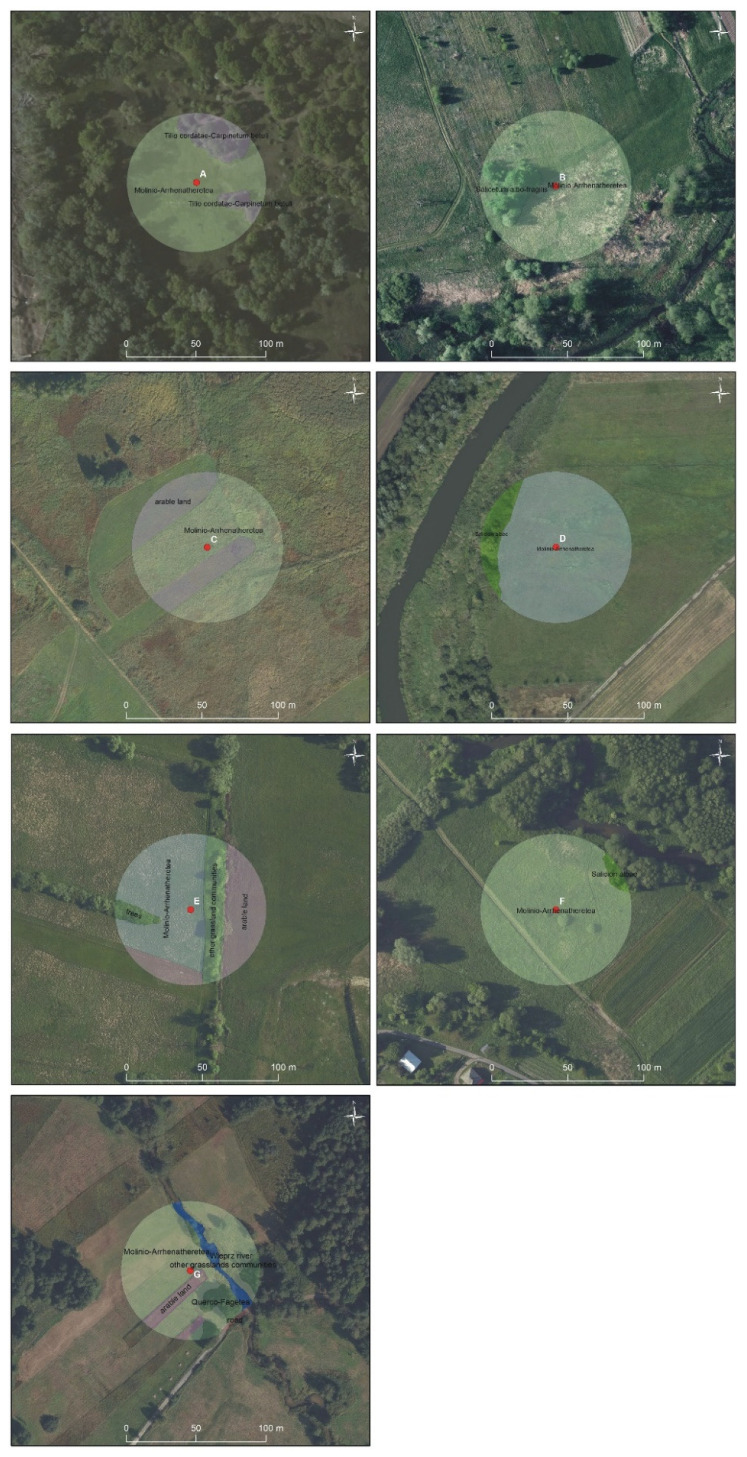
Structure of plant communities in research plots.

Additionally, the vegetation cover in the plots and their surroundings was analyzed. Based on the list of plant species compiled during the field study, the plant communities were classified into associations (in the case of scrubs and shrubs) or classes (meadow vegetation) as in Matuszkiewicz [25]. In the next step, using an orthophotomap downloaded from the WMS Geoportal website [26], the forms of vegetation cover visible in the satellite photos were compared with the plant communities identified previously (Figure A1). Next, using the ArcGIS software (Esri, Redlands, CA, USA) and based on the photointerpretation methods [27,28], the area and percentage share of specific plant communities within a radius of 50 m from the center of the research field were calculated (the diameter of the circle determined in this way corresponded to the length of the transect from which ticks were collected). Additionally, while determining the radius of the studied area, the locomotor abilities of *D. reticulatus* were taken into account [29].

### 2.2. Tick Surveillance

The field studies were carried out between autumn 2018 and autumn 2019. Ticks were collected at regular two-week intervals from the end of February to June (spring period) and from September to mid-November (autumn period) (with modification in the event of unfavorable weather conditions), except the summer months when *D. reticulatus* adults remain inactive in eastern Poland. Ticks were collected during their diurnal peaks of activity, i.e., between 11:00 and 14:00 [30]. The flagging method, which is commonly used for this type of research, was used in accordance with the procedure described by Nowak-Chmura [31]. It consisted in sweeping the vegetation with a white flannel cloth tied to a pole. After covering a distance of approx. 2 m, the flag was turned over, and ticks attached to the cloth were collected using tweezers and placed in a sterile 100-cm^3^ plastic container labeled with information about the plot and date of collection. Each time, ticks were collected in each plot for 30 min.

Additionally, the Data Logger R6030 (Reed Instruments, Wilmington, NC, USA) was used during each tick collection round to measure the weather conditions, air temperature, and relative humidity. The readings were taken at a height of approx. 50 cm, i.e., the average height at which ticks were collected.

In the laboratory, the species, sex, and developmental stage of the ticks were determined using a Zeiss STEMI DV4 stereoscopic microscope (Carl Zeiss Light Microscopy, Göttingen, Germany) and a tick identification key [31]. Next, the ticks were placed in an ULTF freezer (Arctico, Esbjerg, Denmark) at −80 °C.

### 2.3. Statistical Analysis

The statistical tests were chosen based on the characteristics of the distribution of the analyzed variables with the use of the Lilliefors test.

The significance of differences in the number of *D. reticulatus* females and males collected in the research plots was checked with the Wilcoxon test. The differences between the number of ticks that were active in spring and autumn in each study plot were analyzed with the Kruskal-Wallis test. Using one-way ANOVA for independent variables, the differences in the number of active ticks between all research sites were analyzed and the significance of differences in weather conditions between the plots was examined. ANOVA was also used to test the significance of differences in the land cover structure between the study sites. The effect of air temperature and relative humidity on *D. reticulatus* activity was verified with the Sperman rho-correlation test.

A level of significance of *p* ≤ 0.05 was assumed in all statistical tests. The statistical analysis was conducted using Statistica 10PL (StatSoft, TIBCO Software Inc., Palo Alto, CA, USA) software.

## 3. Results

### 3.1. Occurrence and Relative Abundance of Local Dermacentor reticulatus Populations

**Figure 3:**
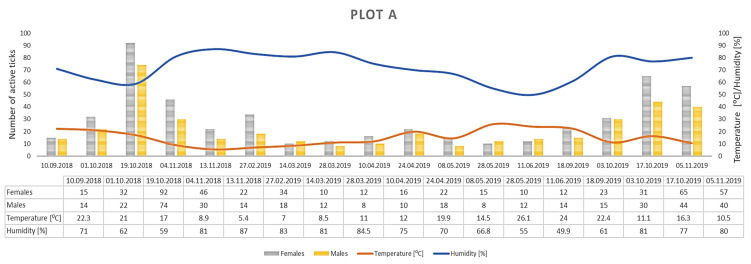
Abundance and seasonal activity of *Dermacentor reticulatus* adults in research plot A and weather conditions prevailing during tick collection. Predominance of active ticks in autumn.

**Figure 4:**
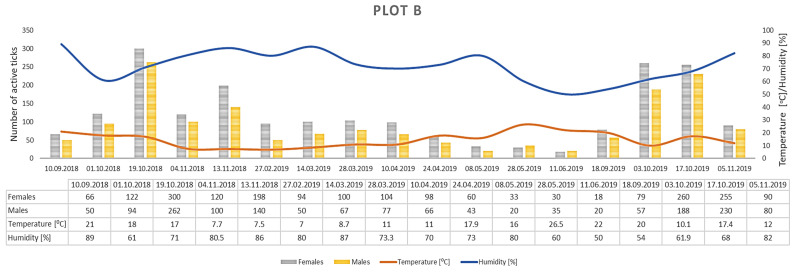
Abundance and seasonal activity of *Dermacentor reticulatus* adults in research plot B and weather conditions prevailing during tick collection. Predominance of active ticks in autumn.

**Figure 5:**
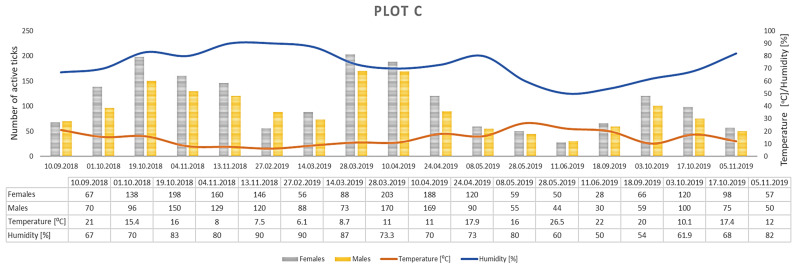
Abundance and seasonal activity of *Dermacentor reticulatus* adults in research plot C and weather conditions prevailing during tick collection. Predominance of active ticks in spring.

**Figure 6:**
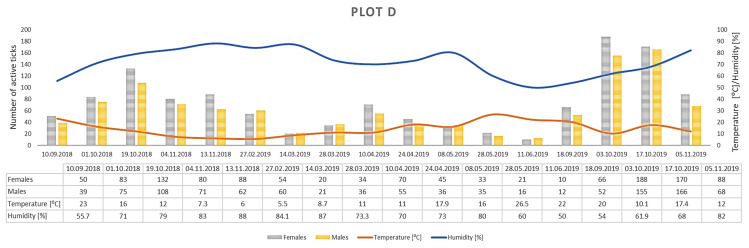
Abundance and seasonal activity of *Dermacentor reticulatus* adults in research plot D and weather conditions prevailing during tick collection. Predominance of active ticks in autumn.

**Figure 7:**
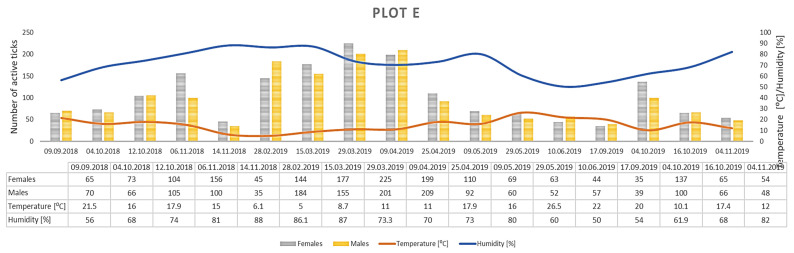
Abundance and seasonal activity of *Dermacentor reticulatus* adults in research plot E and weather conditions prevailing during tick collection. Predominance of active ticks in spring.

**Figure 8:**
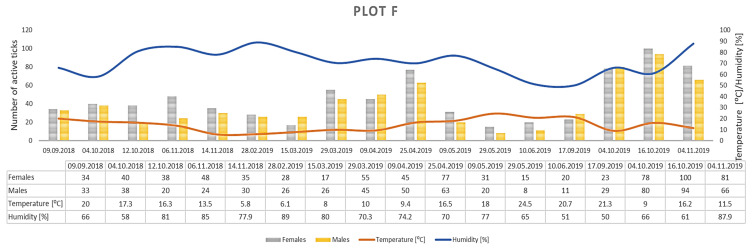
Abundance and seasonal activity of *Dermacentor reticulatus* adults in research plot F and weather conditions prevailing during tick collection. Predominance of active ticks in autumn.

**Figure 9:**
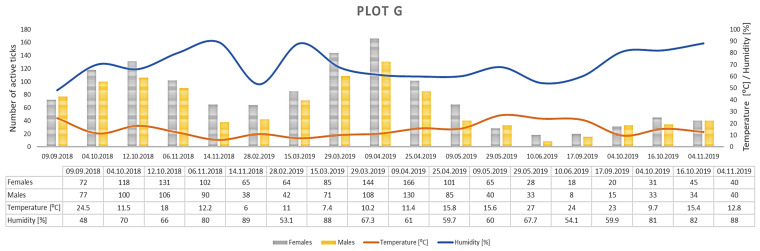
Abundance and seasonal activity of *Dermacentor reticulatus* adults in research plot G and weather conditions prevailing during tick collection. Predominance of active ticks in autumn.

Only *D. reticulatus* adults were collected throughout the study period. In total, 17,389 ticks were collected from all plots, with 9440 females and 7949 males (Figure 3, Figure 4, Figure 5, Figure 6, Figure 7, Figure 8 and Figure 9, Table 2). Higher numbers of active females than males were observed in each plot. This difference was statistically significant in the entire study period (Z = −7.353, *p* < 0.001). Additionally, 98 females, 130 males, and 55 nymphs of *I. ricinus* were collected simultaneously with *D. reticulatus* from all study plots throughout the study period.

The greatest numbers of *D. reticulatus* were collected in plots located in the middle course of the river, i.e., B (on average from 114.3 specimens in spring to 309.7 specimens in autumn per collection), C (on average from 188.8 ticks in spring to 254.8 ticks in autumn per collection), and E (on average from 136.0 specimens in autumn to 255.0 specimens in spring per collection). The lowest numbers of ticks were collected in the research plot located at the confluence of the Wieprz River with the Vistula River (plot A; on average from 28.8 specimens in spring to 76.2 specimens in autumn per collection) (Table 2). Overall, the research plots differed significantly in the number of active ticks (F = 6.928, *p* < 0.01). 

Clear differences in the number of active adult *D. reticulatus* specimens were noted between spring and autumn at each research plot (Figure 3, Figure 4, Figure 5, Figure 6, Figure 7, Figure 8 and Figure 9, Table 2). In plots A, B, D, and F, a higher activity peak was observed in autumn than in spring. The percentage of specimens that were active in autumn in plot D reached 71.0% (Figure 6, Table 2). In plots C and E, greater numbers of ticks were collected in spring (Figure 5 and Figure 7, Table 2).

A statistically significant difference in the number of active ticks between the spring and autumn collection was found in plots A (H = 8.333, *p* = 0.004), B (H = 6.750, *p* = 0.009), D (H = 10.083, *p* = 0.001).

In each of the research sites, the dominant community was represented by the meadow vegetation from the *Molinio-Arrhenatheretea* class (from 57.5% to 97.3% of the area). The greatest numbers of *D. reticulatus*, i.e., on average up to 309.7 individuals, were collected from sites with patches of riparian vegetation (*Salicetum albo-Fragilis*) accompanying the dominant meadow vegetation (Table 2). The structure of the cover of the research plots with the plant communities did not differ significantly (F = 0.00, *p* = 1.000).

### 3.2. Impact of Weather Conditions on Tick Activity

The ticks were collected in a temperature range of 5.0–27.0 °C and relative humidity of 48.0–90.0%. During tick collection, the weather conditions did not differ significantly between the plots (temperature: F = 0.057, *p* = 0.999; relative humidity: F = 0.168, *p* = 0.984).

**Figure 10:**
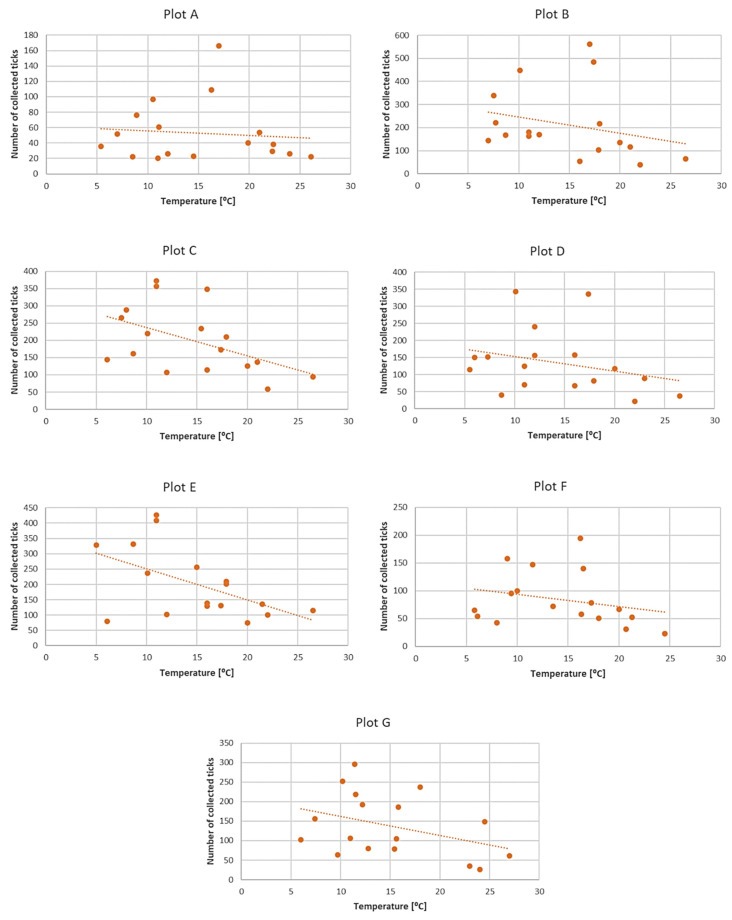
Relationship between air temperature and the number of active *Dermacentor reticulatus* adults in the research plots.

**Figure 11:**
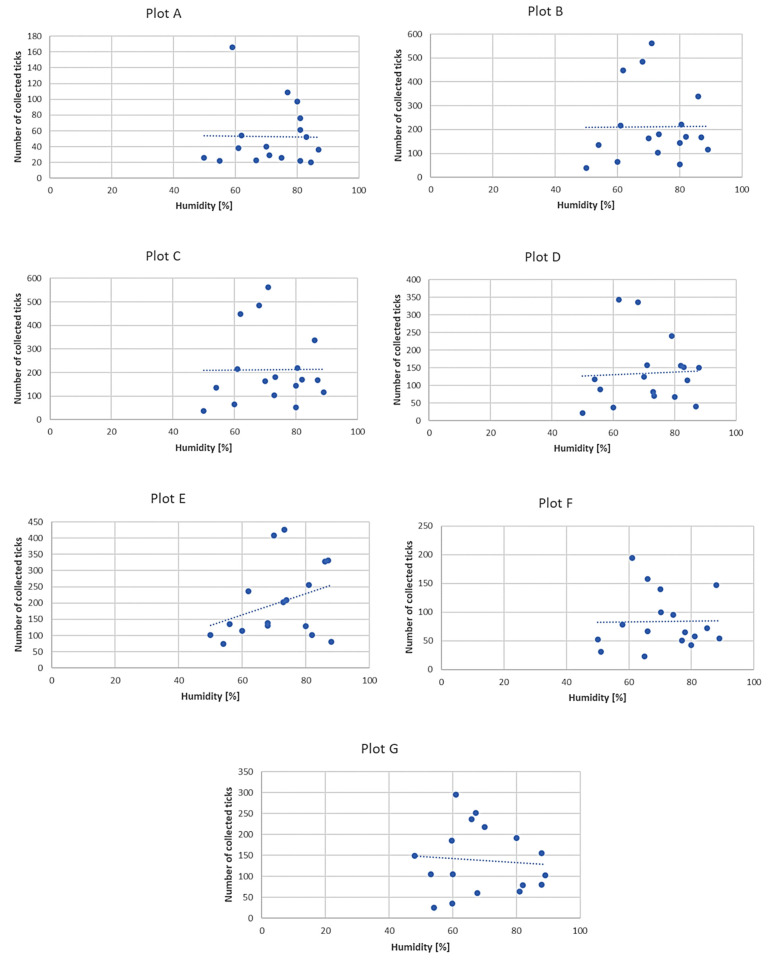
Relationships between relative air humidity and the number of active *Dermacentor reticulatus* adults in the research plots.

In each of the research plots, the number of active ticks declined with an increase in air temperature (Figure 10). No such correlation was observed in the case of relative air humidity, except for plot E, where increased humidity was accompanied by higher activity of *D. reticulatus* specimens (Figure 11) A statistically significant negative correlation was found between the number of active *D. reticulatus* adults and air temperature (r_s_ = −0.304, *p* < 0.001), and there was a statistically non-significant positive correlation between the air relative humidity and the number of active ticks (r_s_ = 0.122, *p* = 0.183).

## 4. Discussion

*Dermacentor reticulatus* ticks exhibit a wide range of tolerance to prevailing weather conditions [14]. They are active in a wide range of temperatures, also in winter months at positive air temperatures and absence of snow cover [32]. During the present study, adult *D. reticulatus* specimens were collected in a temperature range of 5.0–27.0 °C (Table 2, Figure 3, Figure 4, Figure 5, Figure 6, Figure 7, Figure 8, Figure 9 and Figure 10). In other studies conducted in eastern Poland, specimens of this species were collected at a temperature of 34.6 °C [33]. The results indicate a negative correlation between the number of collected ticks and air temperature. This relationship is probably associated with the fact that the greatest numbers of ticks were collected at a lower ambient temperature in autumn in a majority of the plots. The significant effect of temperature on the activity of *D. reticulatus* was also confirmed in other multi-season studies [33,34]. As in the case of other ixodid ticks [35,36,37], the air temperature exerts an impact on the questing activity of *D. reticulatus* [13,30], stimulates the rhythms of seasonal tick activity, and induces diapause [38]. This phenomenon is clearly evident in summer in the temperate climate zone when adults are inactive or only a few ticks are active [31,34]. The increase in air temperature reduces the locomotor activity of *D. reticulatus* [29], which is most probably associated with protection from undesirable water loss. Ticks exposed to substantial water loss die [39,40]. In turn, an increase in the relative humidity in the habitat stimulates locomotor activity in ticks [29].

*D. reticulatus* ticks were collected in every research plot in the present study. The statistical analysis did not show any significant differences in the vegetation structure in the research plots; however, previous studies have revealed these ticks are characterized by high plasticity in relation to the habitats occupied [14,41]. As shown by previous studies conducted in eastern Poland and other countries of this part of the continent, the most abundant *D. reticulatus* populations inhabit lowland areas, which are often covered by unused meadows and fallow land [21,42,43,44,45], in contrast to Western European countries, where this species is collected mostly in forest/semi-forest ecosystems or along the seashore [46,47,48]. In the present study, the predominant plant communities in the analyzed area were fresh semi-natural meadow communities from the class *Molinio-Arrhenatheretea*, which should be regarded as one of the preferred habitats of *D. reticulatus* populations in eastern Poland due to their high abundance.

The *D. reticulatus* populations in the Wieprz River valley as per the current results can be regarded as highly abundant (Figure 3, Figure 4, Figure 5, Figure 6, Figure 7, Figure 8, Figure 9, Figure 10 and Figure 11). Equally large populations of *D. reticulatus* have been reported by Široký et al. [49] in the Czech Republic (investiagators collected up to 222 specimens per hour). An increase in the *D. reticulatus* population size and occurrence range in recent years has been observed in other European countries as well, e.g., in Germany and Slovakia [50,51]. Substantially smaller populations of *D. reticulatus* have been reported from other parts of Poland, e.g., Mierzejewska et al. [18] collected on average 7.4 specimens/100 m^2^ in the central part of the country (Mazowieckie Province), 6.20 specimens/100 m^2^ in western Poland, and 3.04 specimens/100 m^2^ in the north of the country. In the present study, we observed co-occurrence of *D. reticulatus* and *I. ricinus* in the same habitats. This phenomenon has a positive effect on both species, especially in terms of their host-questing activity, feeding period, embryonic development and survival of immature stages [52].

The analyzed *D. reticulatus* populations differed in the number of active specimens in spring and summer (Figure 3, Figure 4, Figure 5, Figure 6, Figure 7, Figure 8, Figure 9, Figure 10 and Figure 11). The autumn peak predominated in plots located in the lower (plots A, B) and upper (plots F, G) course of the river. In the middle course of the river (plots C and E), greater numbers of active ticks were collected in spring (Figure 3, Figure 4, Figure 5, Figure 6, Figure 7, Figure 8 and Figure 9, Table 2). A multiyear study of *D. reticulatus* activity in eastern Poland demonstrated even threefold higher numbers of active ticks in autumn [33,34]. This changed in the 2018/2019 season, i.e., the dominance of the spring peak was observed for the first time in some of the research plots located in the region [21]. It was previously believed that, depending on their geographical range in Europe, *D. reticulatus* exhibit higher activity either in spring (e.g., Russia, France, North-Eastern Poland) or in autumn (Eastern Poland, Central Europe). The seasonality of the activity peaks was ascribed to the adaptation of local populations to thermal conditions and host availability [44,53].

Therefore, especially interesting was the number of active *D. reticulatus* adults observed in plot G (on average 179.8 active specimens in autumn 2018, 148.8 specimens in spring 2019, and 64.5 specimens in autumn 2019) and in plot E (on average 163.8 specimens per collection in autumn 2018, 255.0 specimens in spring 2019, and 136.0 specimens in autumn 2019) (Table 2). The reversal of the patterns of the *D. reticulatus* seasonal activity in eastern Poland has not been fully elucidated. The phenomenon observed may be a result of the drastic decline in the population of the wild boar *Sus scrofa* (regarded as one of the preferred hosts of *D. reticulatus* adults). As estimated by Hunting Clubs, the wild boar population in Lubelskie Province decreased from 7156 to 275 individuals over the last three years [54]. Due to the epidemic of African swine fever spreading in the study area since 2017, wild boars have been subjected to culling in order to stop the spread of the disease. Concurrently, we want to clearly emphasize that the exact determinants of this phenomenon have not been elucidated to date and require further research.

Ecological corridors facilitate undisturbed migration of animals, including small rodents [55], which are the main hosts of juvenile *D. reticulatus* stages, and medium- and large-sized mammals [2,56], i.e., hosts of mature stages. Our studies show that *Apodemus agrarius*, *Microtus arvalis*, and *Myodes glareolus* are the most abundant rodent species in the Wieprz River valley [57]. All these species are commonly regarded as the preferred hosts for *D. reticulatus* larvae and nymphs [47,58]. Furthermore, there are relatively numerous populations of large ungulates *Capreolus capreolus*, *Cervus elaphus*, and *Alces alces* in eastern Poland [54,59]. Previous studies indicate that they are frequently infested by adult *D. reticulatus* [14]. The daily distance covered by the moose and roe deer has been estimated at ca. 2 km and over 4 km, respectively [60]. The mobility of these animals allows ticks to cover certain distances. Moreover, thanks to their biology and ecology, these animals can easily move outside the ecological corridors, thus contributing to the transport of ticks between habitats. The role of birds in the transportation of ticks in these areas is not fully understood. Some studies conducted in northern Poland indicate the possibility of transportation of *D. reticulatus* larvae on birds [61]. Similarly, as reported from other European countries (Norway), blackbirds or thrushes can transport tick larvae (from the genera *Ixodes*, *Hyalomma*, *Dermacentor*) on their bodies [62].

Due to the high abundance of local populations of *D. reticulatus* in the ecological corridor of the Wieprz River and the dominance of meadow vegetation (preferred by this species) along the river valley, migrating animals can be easily attacked by these ticks. In turn, thanks to the long duration of feeding of adult stages on animals [63] and the possibility of overwintering in animal fur [64], ticks can be transported over long distances.

## 5. Conclusions

Adult *Dermacentor reticulatus* ticks occur along the entire ecological corridor in the Wieprz River valley. The local populations of *D. reticulatus* are characterized by predominance of either spring or autumn activity peaks. Given the high abundance of the studied *D. reticulatus* populations, the habitats of the ecological corridor of the Wieprz River can be regarded as preferred habitats of this tick species.

## Figures and Tables

**Table 1:** General characteristics of the mesoregions and the tick collection sites.

Plot/Geographical Coordinates	Mesoregion/Research Plot	Characteristics of the Mesoregion/Research Plots
A 51.540° N, 21.837° E	Middle Vistula Valley 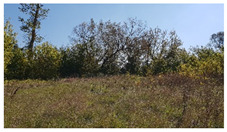	Area adjacent to the Vistula River, 1–1.5 km wide. Chalk substrate. Shrub vegetation in the immediate vicinity of the river. The research plot was established in a clearing surrounded by shrub vegetation with solitary deciduous trees.
B 51.608° N, 22.384° E	Wieprz Glacier Valley 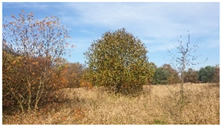	Lower course of the Wieprz River with many meanders. Water sediment substrate. Wetland area. The research plot was established in an unused agricultural meadow adjacent to a mixed forest near the Wieprz River.
C 51.356° N, 22.761° E	Świdnik Plateau 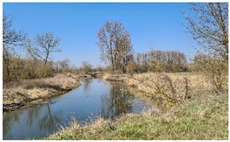	Area located at the confluence of the Wieprz and Bystrzyca Rivers. Flat denudation plain devoid of loess cover. Agricultural region with a low proportion of forests. The research plot was established near the Wieprz River, in an unused meadow surrounded by arable fields and fallow land at a distance of approx. 2 km from a mixed forest.
D 51.180° N, 22.979° E	Dorohusk Basin 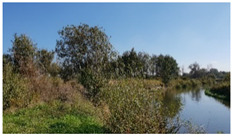	Upland region with Cretaceous rocks in the substrate. Numerous peat bogs and ground depressions are a characteristic feature. The research plot was established in meadow with clearly progressive ecological succession.
E 50.951° N, 23.170° E	Grabowiec Upland 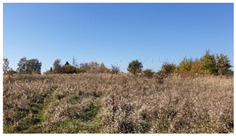	Loess-covered hump formed of Upper Cretaceous rocks. Numerous watercourses joining the Wieprz River. The mesoregion is located on the border of mixed forests and a forest-steppe zone. The maximum altitude is 313 m a.s.l. The research plot was established in an agriculturally unused meadow surrounded by a permanent 5-ha wetland land on one side and a hill escarpment on the other side (approx. 15 m relative height).
F 50.739° N, 23.015° E	Zamość Basin 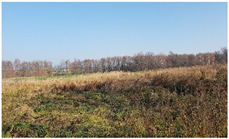	Extensive denudation with chalk marls in the substrate. This plain wetland area is situated approx. 80-m lower (relative height) than the surrounding highlands. The research plot was established in the Wieprz River valley, in an area covered by meadow vegetation.
G 50.584° N, 23.077° E	Middle Roztocze 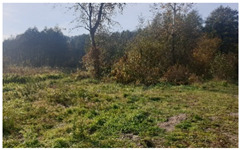	Irregular hills on calcareous substrate without loess cover. Heavily forested area. Annual precipitation sum above the average for the Province. The research plot was established along the bank of the Wieprz River in an area covered by meadow vegetation with shrub islets.

**Table 2:** Structure of plant communities in the research plots, average numbers of *Dermacentor reticulatus* adults collected in the research plots for 30 min, and mean values of air temperature and humidity prevailing at the time of tick collection.

Plot	Structure of Plant Communities in Research Plots [%]	Season *	Mean Value ± SD				
			T [°C]	H [%]	F	M	F + M
A	Cl. *Molinio-Arrhenatheretea* (79.8) Ass. *Tilio cordatae-Carpinetum betuli* (20.2)	Autumn 2018	14.9	72.0	41.4 ± 27.3	30.8 ± 22.4	72.2 ± 49.6
		Spring 2019	15.4	70.6	16.3 ± 7.6	12.5 ± 3.7	28.8 ± 11.1
		Autumn 2019	15.0	74.7	44.0 ± 17.4	32.2 ± 11.8	76.2 ± 29.2
B	Cl. *Molinio-Arrhenatheretea* (86.2) Ass. *Salicetum albo-Fragilis* (13.8)	Autumn 2018	14.2	77.5	161.2 ± 81.1	129.2 ± 72.2	290.4 ± 153.3
		Spring 2019	15.0	71.6	67.1 ± 33.7	47.2 ± 20.2	114.3 ± 53.9
		Autumn 2019	14.8	66.4	171.0 ± 98.7	138.7 ± 80.2	309.7 ± 178.9
C	Cl. *Molinio-Arrhenatheretea* (70.1) Arable land (29.9)	Autumn 2018	13.5	78.0	141.8 ± 42.7	113.0 ± 27.6	254.8 ± 70.3
		Spring 2019	14.9	72.9	99.0 ± 61.4	89.8 ± 49.8	188.8 ± 111.2
		Autumn 2019	14.8	66.4	85.2 ± 25.1	71.0 ± 18.9	156.2 ± 44.0
D	*Molinio-Arrhenatheretea* (89.3) All. *Salicion albae* (10.7)	Autumn 2018	12.8	75.3	86.6 ± 26.3	71.0 ± 22.3	157.6 ± 48.6
		Spring 2019	14.8	72.1	35.8 ± 16.7	33.8 ± 14.9	69.6 ± 31.6
		Autumn 2019	14.9	66.4	128.0 ± 51.9	110.2 ± 50.7	238.2 ± 102.6
E	Cl. *Molinio-Arrhenatheretea* (57.5) Arable land (22.4) Trees (3.0) Other meadow communities (17.1)	Autumn 2018	15.3	73.4	88.6 ± 38.6	75.2 ± 25.4	163.8 ± 64.0
		Spring 2019	14.7	72.4	128.8 ± 63.4	126.2 ± 63.7	255.0 ± 127.1
		Autumn 2019	14.8	66.5	72.7 ± 38.6	63.3 ± 23.3	136.0 ± 61.9
F	Cl. *Molinio-Arrhenatheretea* (97.3) All. *Salicion albae* (2.7)	Autumn 2018	14.5	73.5	39.0 ± 4.9	29.0 ± 6.3	68.0 ± 11.0
		Spring 2019	14.1	72.0	36.0 ± 20.2	31.2 ± 18.3	67.2 ± 38.5
		Autumn 2019	14.5	66.2	70.5 ± 28.6	67.2 ± 24.2	137.7 ± 46.9
G	Cl. *Molinio-Arrhenatheretea* (66.3) Arable lands (8.0) Other meadow communities (6.2) Cl. *Querco-Fagetea* (12.5) Other (7.0)	Autumn 2018	14.4	70.6	97.6 ± 25.5	82.2 ± 24.1	179.8 ± 49.6
		Spring 2019	15.3	63.8	83.8 ± 48.5	64.6 ± 38.6	148.4 ± 87.1
		Autumn 2019	15.2	77.7	34.0 ± 10.6	30.5 ± 9.5	64.5 ± 20.1

* February–June were regarded as the spring period; September-November were regarded as the autumn period (min. temp. at tick collection: 5 °C), SD—standard deviation, T—temperature, h—Humidity, F—Females, M—Males, Cl—syntaxon: class; All—syntaxon: alliance, Ass—syntaxon: association.

## Data Availability

The data that support the findings of this study are available from the corresponding authors, upon reasonable request.
